# Trends in Endometrial Cancer Incidence Among Premenopausal and Postmenopausal Women in the United States Between 2001 and 2021

**DOI:** 10.3390/cancers17061035

**Published:** 2025-03-20

**Authors:** Fangjian Guo, Victor Adekanmbi, Christine D. Hsu, Thao N. Hoang, Pamela T. Soliman, Jacques G. Baillargeon, Abbey B. Berenson

**Affiliations:** 1 Department of Obstetrics & Gynecology, The University of Texas Medical Branch at Galveston, Galveston, TX 77555, USA; viadekan@utmb.edu (V.A.); cdhsu@utmb.edu (C.D.H.); thnhoang@utmb.edu (T.N.H.); abberens@utmb.edu (A.B.B.); 2 Center for Interdisciplinary Research in Women’s Health, The University of Texas Medical Branch at Galveston, Galveston, TX 77555, USA; 3 Department of Gynecologic Oncology and Reproductive Medicine, University of Texas MD Anderson Cancer Center, Houston, TX 77230, USA; psoliman@mdanderson.org; 4 Department of Epidemiology, School of Public and Population Health, The University of Texas Medical Branch at Galveston, Galveston, TX 77555, USA; jbaillar@utmb.edu

**Keywords:** endometrial cancer, incidence, hysterectomy, COVID-19, trend

## Abstract

Background: Endometrial cancer incidence has been rising in the United States. We assessed trends in endometrial cancer incidence among both premenopausal and postmenopausal women in the US from 2001 to 2021. We also compared the incidence during 2019–2021 to assess the impact of the COVID-19 pandemic. Methods: We used data from the United States Cancer Statistics 2001–2021 database to assess the incidence trends among adult females. Endometrial cancer incidence was corrected for hysterectomy prevalence and age, adjusted to the 2000 US standard population. Results: From 2001 to 2021, the incidence of endometrial cancer rose from 86.8 cases to 113.8 cases per 1,000,000 persons among women aged 20–49 years (APC 1.5, 95% CI 1.2–1.8), while in women 70 and older, it increased from 1326.4 cases to 1339.4 cases per 1,000,000 persons (APC 0.3, 95% CI 0.1–0.6). The incidence has recently decreased among women aged 50–69 years (APC from 2001 to 2016 0.3, 95% CI 0.1–0.9; APC from 2016 to 2021 −1.3, 95% CI −2.2–−0.3). Endometrial cancer incidence sharply increased from 2001 to 2021 among non-Hispanic Blacks, non-Hispanic Asians or Pacific Islanders, and women in the South. Endometrial cancer incidence sharply decreased from 2019 to 2020, and the proportion of metastatic cancer at diagnosis increased across all age groups. In 2021, the incidence returned to 2019 levels. Conclusions: Endometrial cancer incidence rates are rising, particularly among premenopausal women. During the beginning of the COVID-19 pandemic, the incidence rates decreased, but the proportion of metastatic cancer increased.

## Simple Summary

The aim of this study was to assess endometrial cancer incidence in both premenopausal and postmenopausal women in the US from 2001 to 2021 using data for US adult females from the United States Cancer Statistics 2001–2021 database. From 2001 to 2021, endometrial cancer incidence increased in those aged 20–49 years and in those aged 70+ years. Endometrial cancer incidence has decreased in recent years (from 2016 to 2021) among adults aged 50–69 years. The incidence rate of endometrial cancer sharply decreased from 2019 to 2020 in all age groups, and the proportion of metastatic cancer increased across all age groups. In 2021, the incidence returned to 2019 levels. In conclusion, there were increasing trends in endometrial cancer among premenopausal women between 2001 and 2021. During the beginning of the COVID-19 pandemic, the incidence rates decreased, but the proportion of metastatic cancer increased.

## 1. Introduction

Uterine cancer is the most common gynecologic malignancy in the United States [1,2]. Uterine cancer includes a broader category of cancers that can affect various parts of the uterus, including the endometrium and myometrium. The majority of uterine cancer cases involve the endometrium, and endometrial cancer cases account for over 90% of all uterine cancer cases [1,2]. Endometrial cancer typically develops when cells in the endometrium undergo abnormal changes and begin to multiply uncontrollably. While a variety of hormonal, genetic, and environmental factors can cause uterine cancer, the most prominent risk factor for this cancer is exposure to endogenous or exogenous estrogen without sufficient opposing progesterone. Conditions such as obesity, polycystic ovary syndrome, and hormone replacement therapy have also been associated with an increased risk of uterine cancer [1,3,4,5]. Among premenopausal women, other factors, including early menarche and delayed childbearing, may also contribute to the increased incidence [4,6,7]. Endometrial cancer subtypes exhibit distinct risk profiles and molecular characteristics [8]. Type I tumors are associated with obesity and estrogen excess, while Type II tumors are more prevalent in non-obese women. *PTEN*, *FGFR2*, *ARID1A*, *CTNNB1*, *PIK3CA*, *PIK3R1*, and *KRAS* mutations are frequent in Type I (primarily endometrioid) tumors, whereas *TP53*, *PIK3CA*, and *PPP2R1A* mutations are often found in Type II (mostly serous) tumors [8,9,10,11].

The widespread use of diagnostic tools such as transvaginal ultrasound and endometrial sampling in patients with irregular vaginal bleeding has improved the early detection of uterine abnormalities [12,13], including precancerous lesions and early-stage cancers. As a result, these advancements may have contributed to the reported increase in endometrial cancer incidence rates [14]. Endometrial cancer poses a substantial burden on both the patient and the healthcare system. However, data regarding updated trends in the incidence of endometrial cancer are limited, and recent studies on endometrial cancer incidence were not corrected for hysterectomy prevalence [14]. A recent report on incidence rates that account for hysterectomy prevalence spanned the period from 2004 to 2008 in the United States [15]. Therefore, we examined the shifting patterns of endometrial cancer incidence among pre- and postmenopausal women in the United States from 2001 to 2021. In addition, we compared endometrial cancer incidence between 2019, 2020, and 2021, considering the public’s reduced access and inclination to use healthcare services in the early phase of the COVID-19 pandemic [16].

## 2. Methods

### 2.1. Study Design

This study was conducted from 1 September 2022 to 24 November 2024, using data from the United States Cancer Statistics (USCS) database [17], which contains cancer incidence and population data for all 50 states and the District of Columbia. Hospitals, physicians, and laboratories across the nation report data on patient demographic and tumor characteristics to central cancer registries supported by the CDC and NCI, i.e., the CDC’s National Program for Cancer Registries (NPCR) and the NCI’s Surveillance, Epidemiology, and End Results (SEER) Program. The NPCR and SEER Incidence—USCS public use database for 2001–2021 covers the adult female population in the US; from this, we analyzed 914,429 cases of new-onset endometrial cancer from 2001 to 2021. This study was exempt from a full board review by the Institutional Review Board of The University of Texas Medical Branch at Galveston, Galveston, TX. This report follows the STROBE reporting guidelines.

The information collected about each endometrial cancer case included the patient’s demographic characteristics and date of cancer diagnosis. Uterine cancer cases were defined as microscopically confirmed malignancies of the following sites: International Classification of Diseases for Oncology, Third Edition (ICD-O-3) site codes C54.0 (isthmus uteri), C54.1 (endometrium), C54.2 (myometrium), C54.3 (fundus uteri), C54.8 (overlapping lesion of corpus uteri), C54.9 (corpus uteri), and C55.9 (uterus, not otherwise specified). Cases identified solely by autopsy or death certificate were excluded. Only cases classified as malignant under ICD-O-2 and ICD-O-3 were included in this report. Endometrial cancer cases were defined as uterine cancer with the following ICD-O-3 histologic types: endometrioid carcinomas (8380) and other carcinomas (8000–8379, 8381–8790, 8981, 9700–9701). The histologic types were classified as follows: Type I [adenocarcinoma, NOS (8140), adenocarcinoma tubular (8210, 8211), papillary adenocarcinoma (8260, 8262, 8263), endometrioid (8380, 8381, 8382, 8383), mucinous adenocarcinoma (8480, 8481, 8482), adenocarcinoma with squamous/adenosquamous carcinoma (8560, 8570)] and Type II [serous/papillary serous (8441, 8460, 8461), clear cell (8310), and other (small-cell carcinoma (8041), squamous cell (8050, 8070, 8071, 8072, 8076), mixed-cell adenocarcinoma (8323), malignant mixed mullerian tumors (8950, 8951, 8980, 8981, 8982)]. We categorized the cases by histologic grade, as follows: low-risk carcinomas (well differentiated/Grade I and moderately differentiated/Grade II in 2001–2017; Grade 1 and Grade 2 in 2018–2020) and high-risk carcinomas (poorly differentiated/Grade III and undifferentiated/anaplastic/Grade IV in 2001–2017; Grade 3 in 2018–2020). We considered three stages of endometrial cancer at the time of diagnosis: localized, regional, and distant/metastatic.

For endometrial cancer incidence analysis, data were grouped into the following three age categories: women aged 20–49 years (premenopausal), 50–69 years, and 70 years or older. We included the following four regions of residence: the Northeast, Midwest, South, and West. Our analysis included data on race (non-Hispanic White, non-Hispanic Black, non-Hispanic American Indian/Alaska Native, and non-Hispanic Asian or Pacific Islander) and ethnicity (Hispanic or non-Hispanic). Hispanic ethnicity was identified for all cancer cases using the North American Association of Central Cancer Registries (NAACCR) Hispanic/Latino Identification Algorithm (NHIA) [18].

Behavioral Risk Factor Surveillance System (BRFSS) data from 2000 to 2022 were used to estimate hysterectomy prevalence among adult females in the United States. Questions about hysterectomy were posed in even-numbered years from 2000 to 2022 for all jurisdictions. The BRFSS is an ongoing telephone (landline and cellular) survey that collects data from over 400,000 adult respondents in the United States each year, spanning all 50 states, the District of Columbia, and US territories. The survey covers a wide range of topics related to health-risk behaviors, chronic health conditions, and the use of preventive services. We used data from the BRFSS for hysterectomy prevalence estimation, as it had a larger sample size and allowed for estimations for subpopulations when compared to the data from the National Health Interview Survey [19]. The hysterectomy question in the BRFSS is as follows: “Have you had a hysterectomy?” We recoded pregnant respondents at the time of survey administration as not having a hysterectomy.

### 2.2. Statistical Analysis

All analyses were carried out using the SEER*Stat statistical software package (version 8.4.4), SAS software version 9.4 (SAS Institute; Carey, NC, USA), and the Joinpoint Regression Analysis program, version 5.3.0.0 [20]. Statistical significances were determined as two-sided *p* values < 0.05. Endometrial cancer incidence was adjusted for hysterectomy prevalence and age adjusted to the 2000 US standard population. As hysterectomy data were only available on even-numbered years from 2000 to 2022 for all jurisdictions, we estimated the hysterectomy prevalence for odd-numbered years by averaging their adjacent even-numbered years using similar methods described elsewhere [21,22]. For example, we estimated the hysterectomy prevalence in 2021 by averaging the prevalence in 2020 and 2022. We included stratification, clustering, and weighting for BRFSS data to account for its complex sampling design. To calculate hysterectomy-adjusted endometrial cancer incidence, we estimated hysterectomy prevalence in the following age groups: 20–29, 30–39, 40–49, 50–59, 60–69, 70–79, and 80+, and in subgroups by race/ethnicity and region of residence. To determine the population-at-risk denominator for endometrial cancer, we first removed women who had undergone hysterectomy and then added back women who were diagnosed with endometrial cancer within that year. Incidence rates of endometrial cancer were expressed as cases per 1,000,000 persons and age-adjusted to the 2000 US standard population. We used the Tiwari method to determine the confidence intervals [23]. Joinpoint regression models [24] were applied to annual incidence data from 2001 to 2021 using the NCI’s Joinpoint Regression Analysis program (version 5.3.0.0) [20]. This analysis program identified joinpoints (calendar years) where annual percentage changes (APCs) significantly shifted, selecting the best-fitting log-linear regression model with the fewest necessary joinpoints. APC was calculated using the formula (exp[β] − 1) × 100, where β represents the regression coefficient obtained from a least-squares regression of the natural logarithm of rates against the calendar year. The statistical significance of APCs and differences between APCs were evaluated using Kleinbaum’s methods [25]. Subgroup analyses were conducted for various age groups and races/ethnicities. We also compared the endometrial cancer incidence between 2019, 2020, and 2021 because the COVID-19 pandemic affected the public’s ability and desire to use healthcare services, which resulted in postponed and decreased cancer screenings and diagnoses, causing a significant temporary decrease in incidence rates [16]. Statistics were not reported in cases where the count fell below 16, a measure taken to safeguard patient confidentiality by minimizing the risk of identity disclosure. Additionally, this practice was adopted due to the inherent unreliability of rates calculated from a small number of cases [26]. Unreported statistics were handled as missing values.

## 3. Results

Between 2001 and 2021, 914,429 adult females were diagnosed with new-onset endometrial cancer. These included 79,271 Hispanics, 703,966 non-Hispanic Whites, 86,751 non-Hispanic Blacks, 5336 non-Hispanic American Indian/Alaska Natives, and 32,909 non-Hispanic Asians or Pacific Islanders.

**Figure 1:**
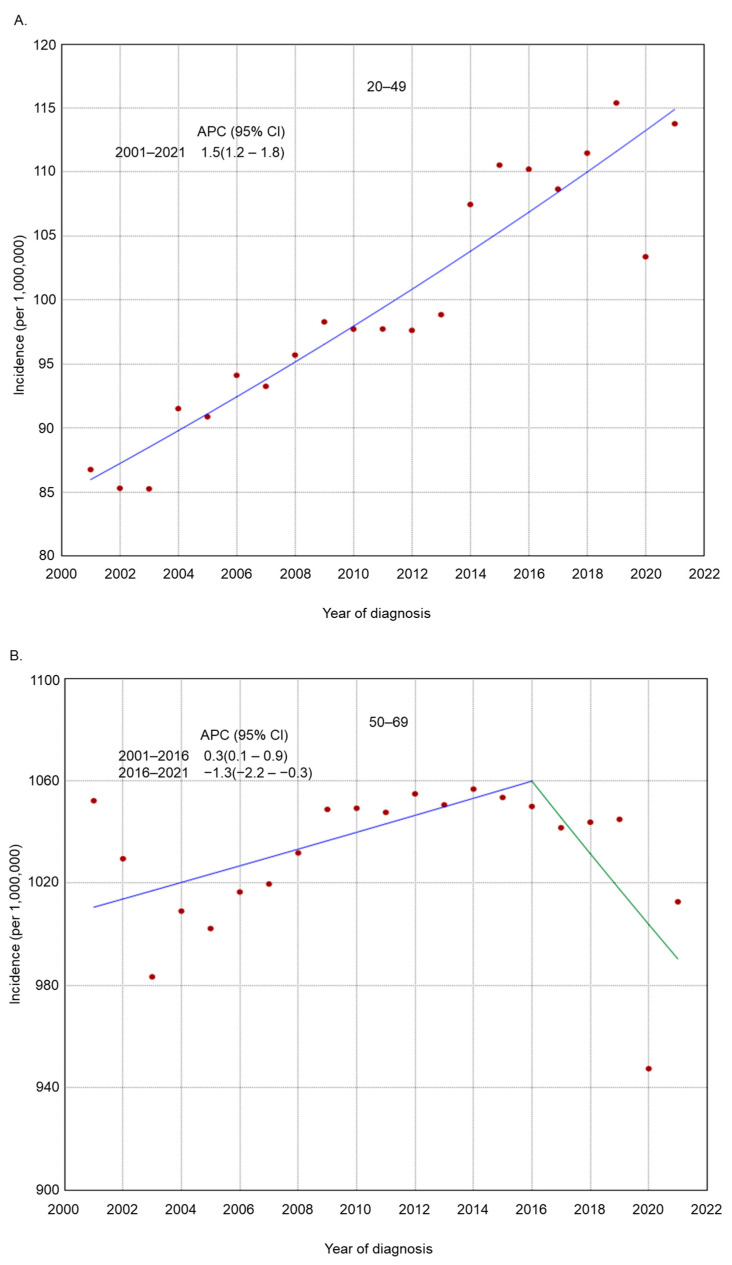

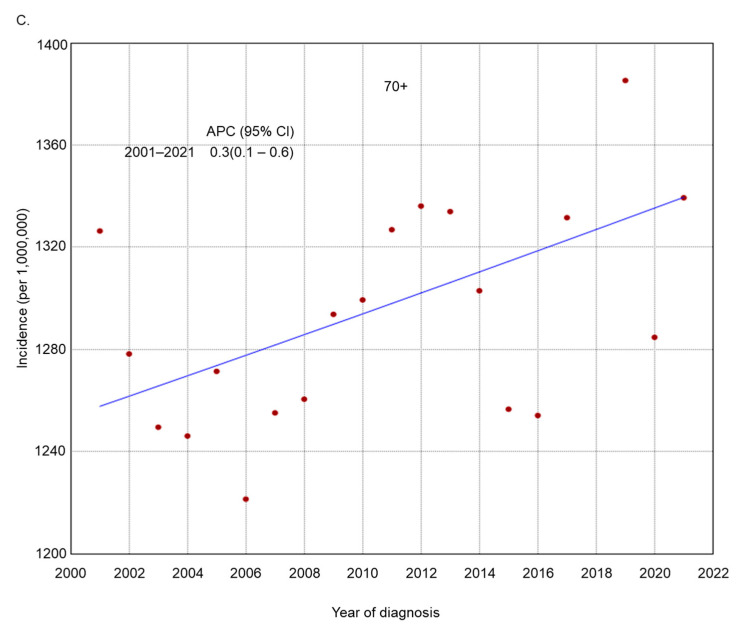
Adjusted incidence rates of endometrial cancer in the United States from 2001 to 2021 among adult women belonging to the following age groups: (**A**) 20–49 years; (**B**) 50–69 years; and (**C**) 70+ years.

From 2001 to 2021, the incidence of endometrial cancer increased from 86.8 cases to 113.8 cases per 1,000,000 persons (APC 1.5, 95% CI 1.2–1.8) in women aged 20–49 years and from 1326.4 cases to 1339.4 cases per 1,000,000 persons (APC 0.3, 95% CI 0.1–0.6) in women aged 70+ years (Figure 1). The Joinpoint analyses from 2001 to 2021 revealed that there was only one joinpoint for the trends in the rate of endometrial cancer incidence among women aged 50–69 years (APC from 2001 to 2016 0.3, 95% CI 0.1–0.9; APC from 2016 to 2021 −1.3, 95% CI −2.2–−0.3).

**Figure 2:**
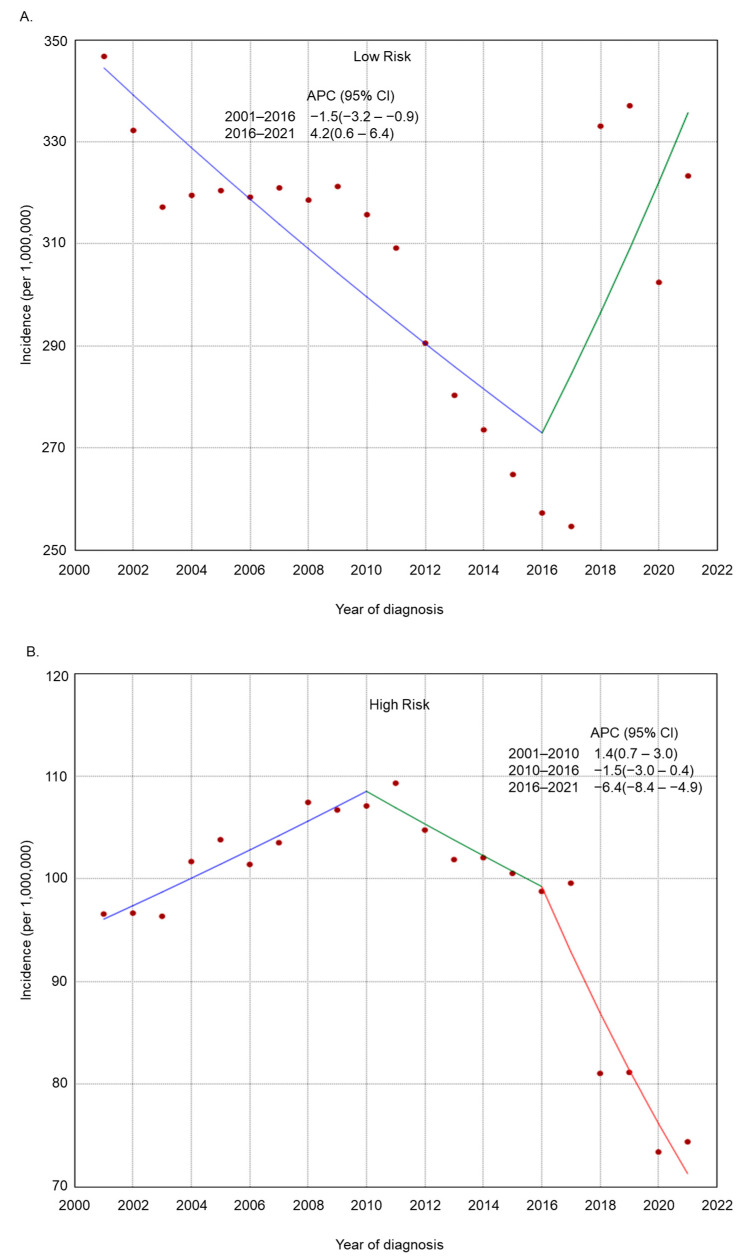
Adjusted incidence rates of endometrial cancer among adult women in the United States from 2001 to 2021 by histologic grade category: (**A**) low risk and (**B**) high risk.

**Figure 3:**
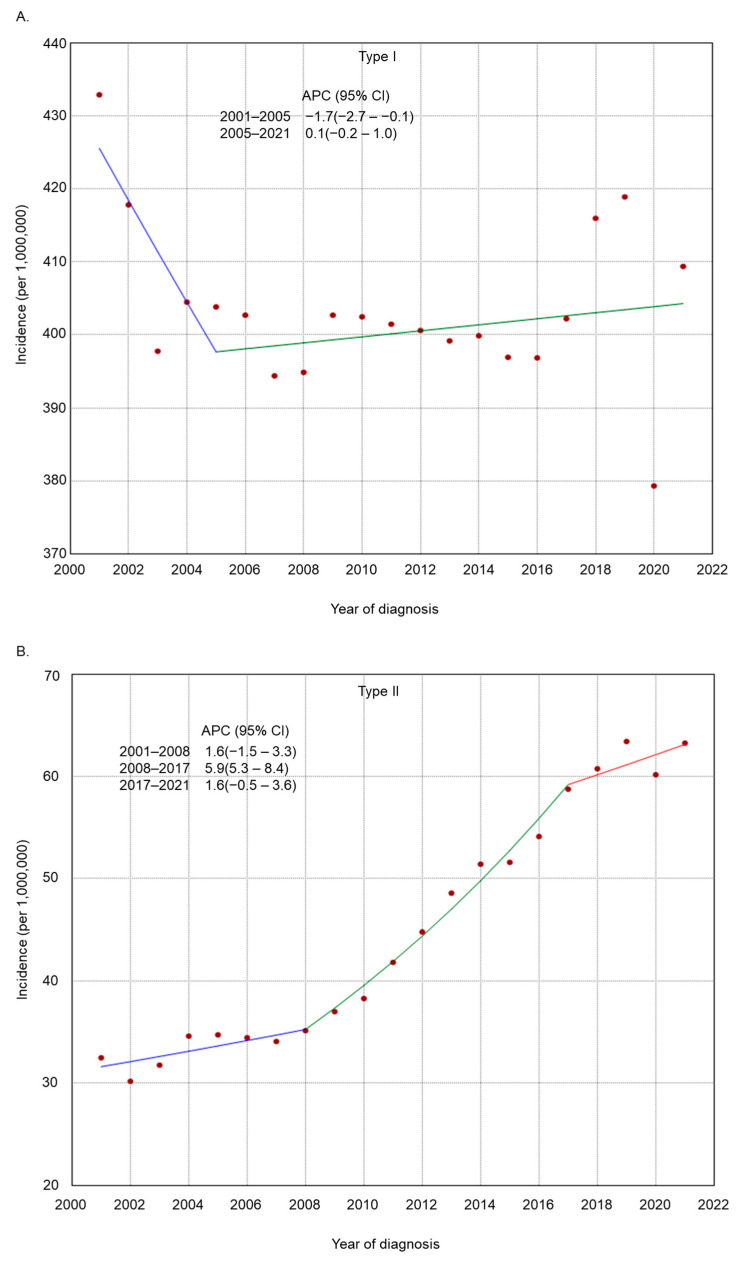
Adjusted incidence rates of endometrial cancer among adult women in the United States from 2001 to 2021 by histologic type: (**A**) Type I and (**B**) Type II.

**Figure 4:**
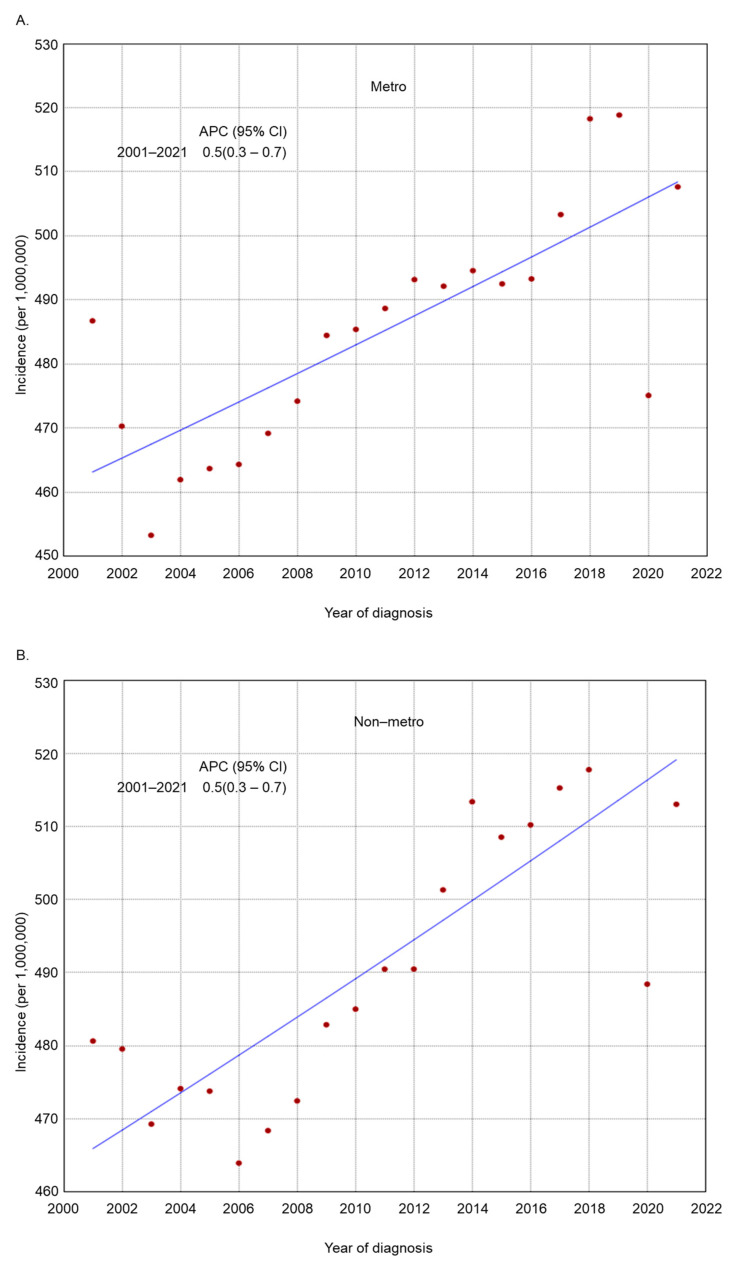
Adjusted incidence rates of endometrial cancer among adult women in the United States from 2001 to 2021 by residence type: (**A**) urban and (**B**) rural.

**Figure 5:**
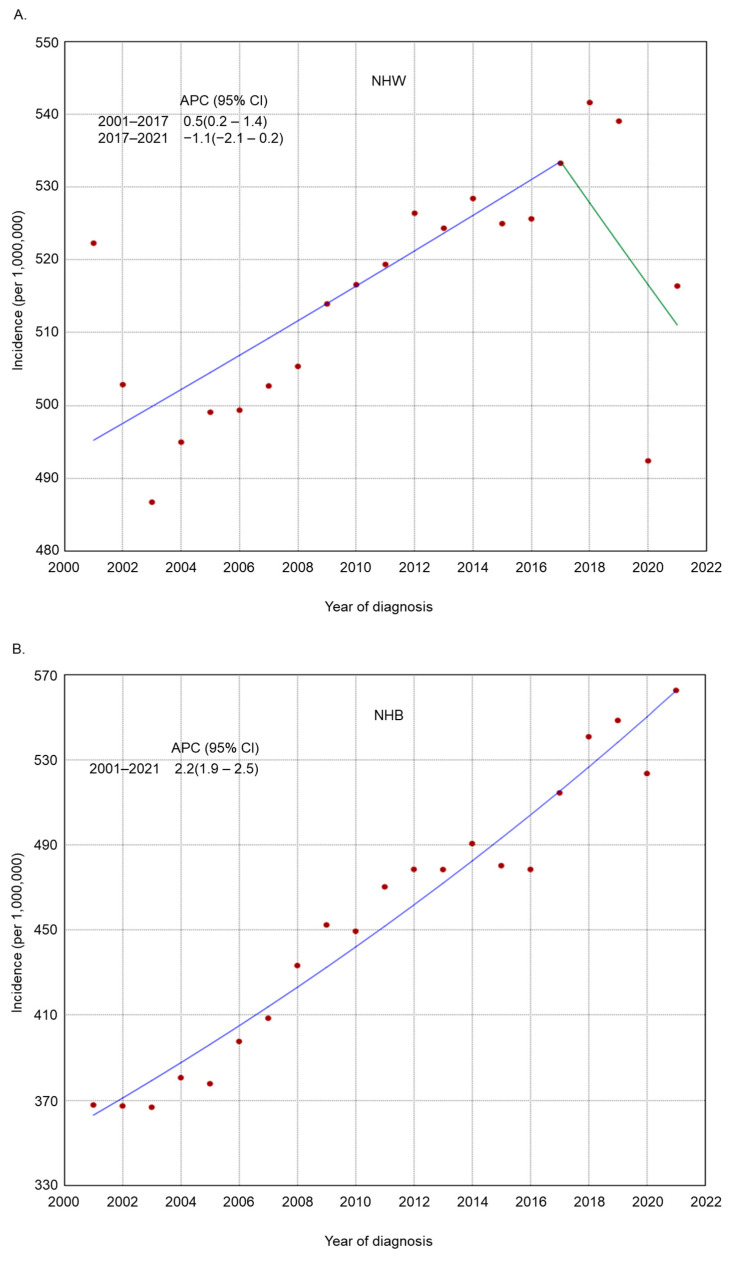

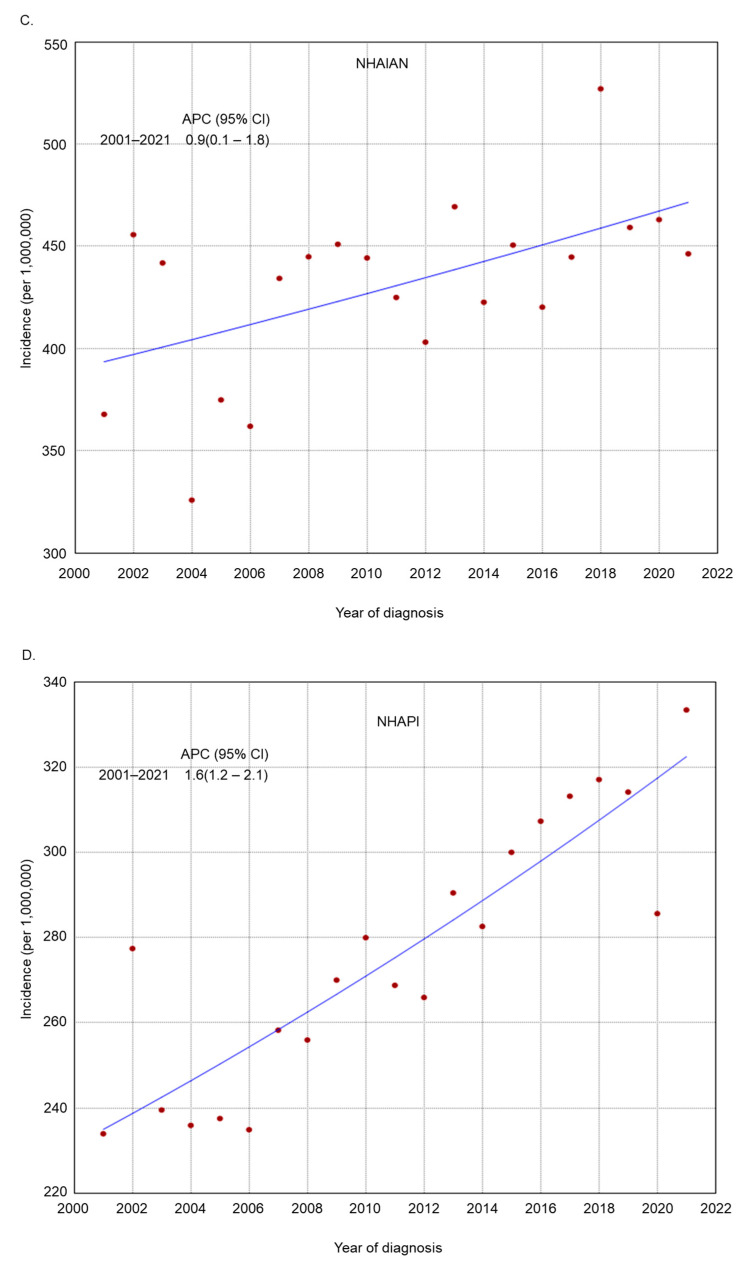

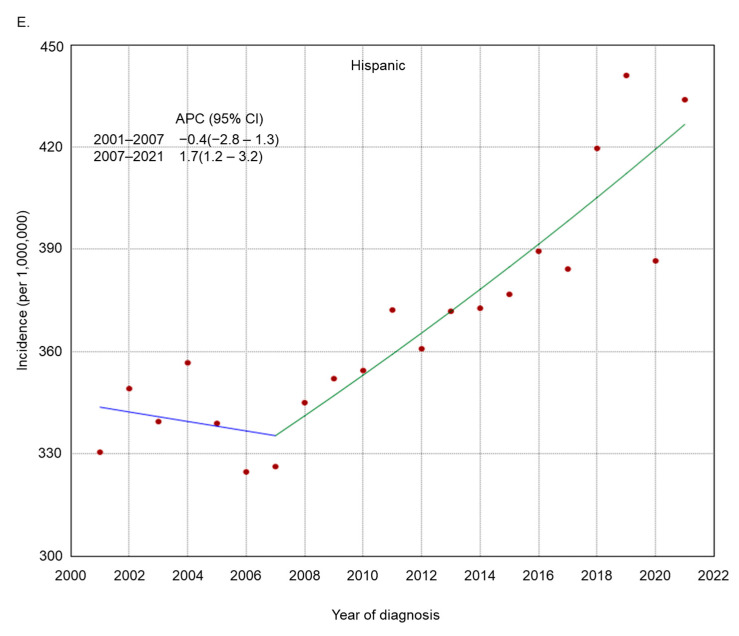
Adjusted incidence rates of endometrial cancer among adult women in the United States from 2001 to 2021 by race/ethnicity. (**A**) NHW; (**B**) NHB; (**C**) NHAIAN; (**D**) NHAPI; (**E**) Hispanic. NHW: non-Hispanic White; NHB: non-Hispanic Black; NHAIAN: non-Hispanic American Indian/Alaska Native; NHAPI: non-Hispanic Asian or Pacific Islander.

**Figure 6:**
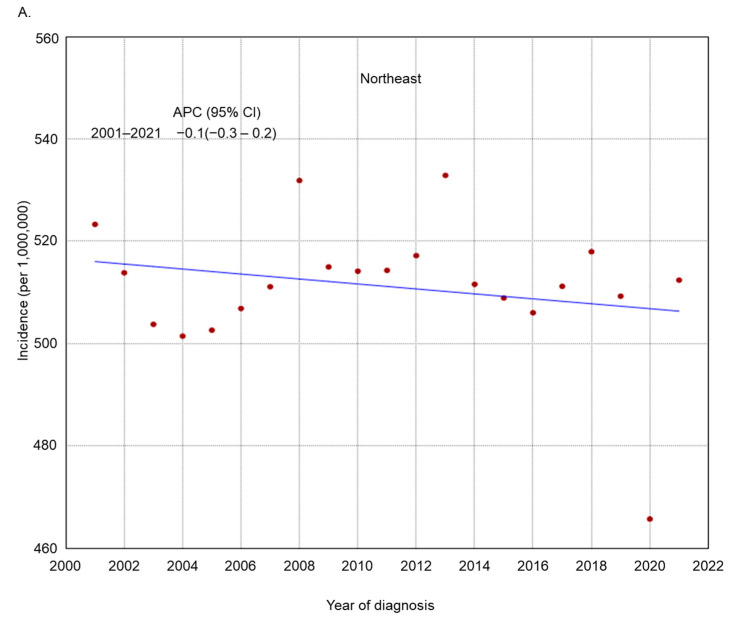

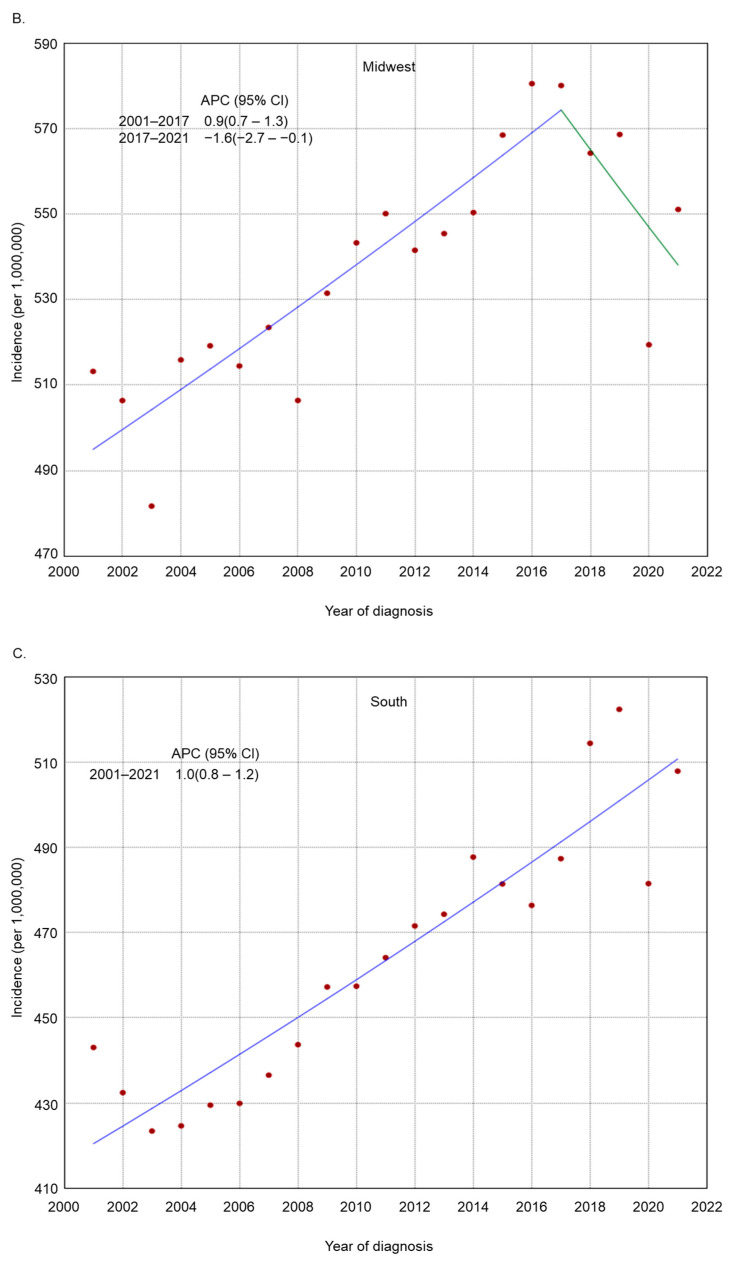

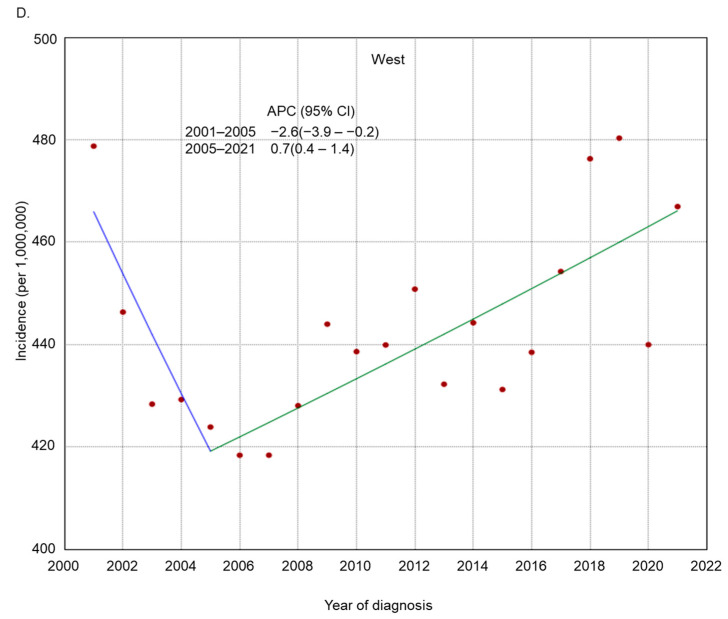
Adjusted incidence rates of endometrial cancer among adult women in the United States from 2001 to 2021 by region of residence: (**A**) Northeast; (**B**) Midwest; (**C**) South; (**D**) West.

The incidence rate of endometrial cancer increased across several subgroups based on histologic grade, histologic type, residence type, race/ethnicity, and region of residence (Figure 2, Figure 3, Figure 4, Figure 5 and Figure 6). Endometrial cancer incidence sharply increased from 2001 to 2021 among non-Hispanic Blacks (APC 2.2, 95% CI 1.9–2.5), non-Hispanic Asians or Pacific Islanders (APC 1.6, 95% CI 1.2–2.1), and women in the South (APC 1.0, 95% CI 0.8–1.2). Recently, high-risk endometrial cancer incidence has declined from 2016 to 2021 (APC −6.4, 95% CI −8.4–−4.9). The incidence of endometrial cancer declined from 2017 to 2021 (APC −1.1, 95% CI −2.1–−0.2) among non-Hispanic Whites and from 2017 to 2021 (APC −1.6, 95% CI −2.7–−0.1) among women in the Midwest. The incidence of endometrial cancer among women in the Northeast has remained relatively steady recently (APC from 2016 to 2021 −0.1, 95% CI −0.3–0.2).

**Figure 7:**
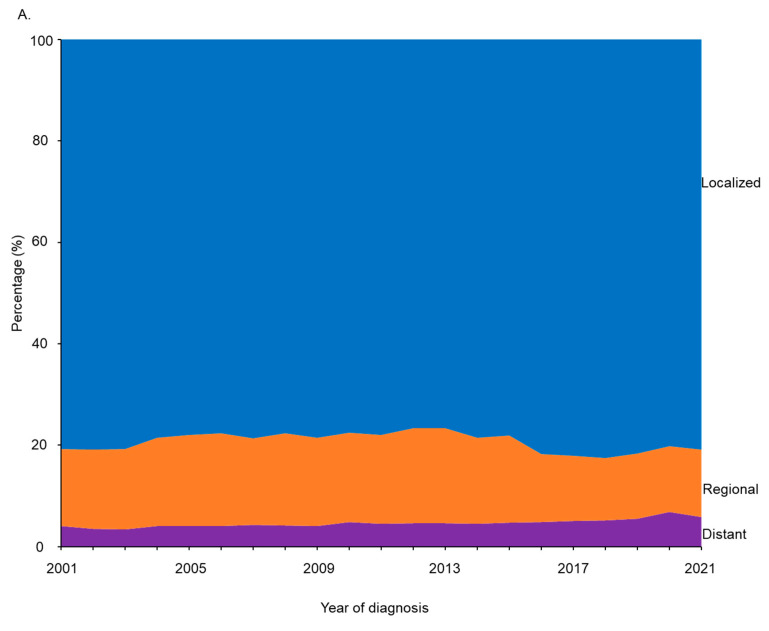

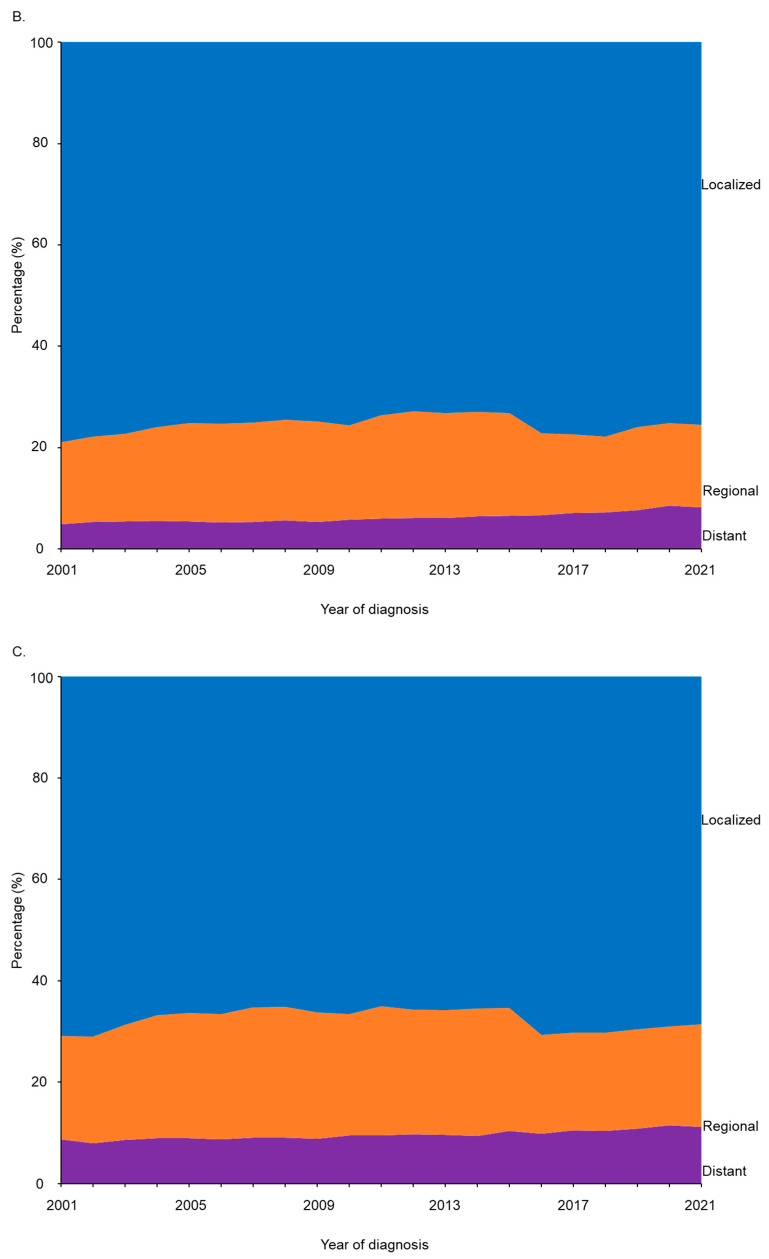
Percentages of stages at the time of diagnosis of endometrial cancer among adult women in the United States from 2001 to 2021 by age group: (**A**) 20–49 years old; (**B**) 50–69 years old; (**C**) 70+ years old.

There were 54,571 cases of endometrial cancer in 2019, 49,917 cases in 2020, and 54,225 cases in 2021 among adult women 20 years and older. The incidence rates of endometrial cancer decreased sharply from 2019 to 2020 in all age groups (Table 1), although the proportion of metastatic cancer among these cases increased across all age groups (Figure 7). From 2019 to 2020, the incidence rate of endometrial cancer decreased from 115.4 cases to 103.4 cases per 1,000,000 persons among females aged 20–49 years, from 1045.0 cases to 947.6 cases per 1,000,000 persons among females aged 50–69 years, and from 1385.3 cases to 1284.8 cases per 1,000,000 persons among females aged 70 years and older. The proportion of distant/metastatic endometrial cancer at the time of diagnosis among these cases increased from 5.2% in 2019 to 6.5% in 2020 among females aged 20–49 years, from 7.5% to 8.3% among females aged 50–69 years, and from 10.3% to 11.0% among females aged 70 years and older. In 2021, the incidence rates of endometrial cancer returned to 2019 levels.

## 4. Discussion

Our study examined the trends in endometrial cancer incidence among premenopausal and postmenopausal women in the United States from 2001 to 2021. The findings reveal a significant increase in endometrial cancer incidence among women aged 20–49 years and 70+ years. Among women aged 50–69 years, endometrial cancer incidence decreased from 2016 to 2021. These divergent age-specific analyses necessitate further research to understand the underlying causes. The overall increase in incidence rates aligns with previously observed trends [2,14,27,28], highlighting the growing burden of endometrial cancer across different demographic groups. The increasing incidence of endometrial cancers among premenopausal women is particularly concerning, as this group now represents a significant portion of all diagnosed endometrial cancer cases. Given this trend, fertility-sparing treatment strategies, such as conservative management with progestin therapy and fertility preservation through oocyte vitrification, should be considered for women of reproductive age with early-stage, low-grade endometrioid subtypes [29,30,31,32,33,34,35]. Additionally, psychosocial counseling can be used to mitigate the stressors and emotional challenges involved in making complex decisions about fertility preservation and cancer treatment options [36,37].

Obesity has been associated with the development of many different types of cancer [38]. More specifically, it has become the most significant and prevalent risk factor for endometrial cancer [1,39], with about 60% of endometrial cancers in the US linked to obesity [1,3,38,40,41]. The substantial increase in the prevalence of obesity in the United States over the last few decades [42] may, in part, explain the increased incidence of endometrial cancer. Obesity contributes to endometrial cancer risk through various mechanisms, including alterations in hormonal profiles, chronic inflammation, and insulin resistance, all of which promote abnormal endometrial cell proliferation and carcinogenesis. Obesity is linked to excess body fat, which leads to hormonal imbalances and elevation in the levels of estrogen that go unopposed by progesterone. This can then lead to endometrial hyperplasia and subsequently cancer [1,43,44,45,46]. It is important to note that high-risk histologic carcinomas are not linked to obesity. These types of endometrial cancers represent only 15% of cases, but contribute up to 50% of cancer-related deaths [47,48,49]. The reasons behind this trend remain unclear but may involve a complex interplay of biological, socioeconomic, and healthcare access factors that warrant further investigation.

While obesity remains the most significant and prevalent risk factor for endometrial cancer, changed reproductive patterns and delayed childbearing may also contribute to the increasing incidence of endometrial cancer among premenopausal women [50,51]. A comprehensive meta-analysis involving 69,681 participants found a non-linear inverse correlation between the number of pregnancies a woman has had and the risk of endometrial cancer [52]. This association could potentially be explained by the increase in progesterone, which counteracts estrogen-driven endometrial proliferation and reduces the risk of malignant transformation. Additionally, early menarche and family planning practices such as decreased parity and older maternal age at first birth, both of which have become more common in modern societies, could also contribute to shifts in endometrial cancer epidemiology [5,52,53,54]. Moreover, a decline in hysterectomies over recent decades could potentially contribute to the increased incidence of endometrial cancer [55,56].

The widespread adoption of diagnostic tools for the early detection of endometrial cancer in recent years has likely contributed to the observed increase in incidence rate [12,13]. Transvaginal ultrasonography is a highly effective imaging modality which enables clinicians to assess endometrial thickness and detect abnormalities with high resolution and accuracy. When combined with endometrial biopsy, this approach facilitates early diagnosis and improves patient outcomes by identifying malignancies at an earlier, more treatable stage. Continued research efforts and investments in healthcare technology are needed to address these challenges and to implement effective strategies for the prevention, early detection, and treatment of endometrial cancer [57,58,59,60].

Our findings regarding the trends in endometrial cancer align with the results of previous studies [2,14,27,28]. We found overall increasing trends in endometrial cancer incidence during the last two decades. We observed similar racial/ethnic trends as those previously reported without adjustment for hysterectomy prevalence [14]. Non-Hispanic Blacks exhibited lower incidence rates compared to those of non-Hispanic Whites in the first decade; however, rates in non-Hispanics increased sharply and surpassed those in non-Hispanic Whites in recent years. This finding contradicts the earlier assumption that adjusting for hysterectomy prevalence by racial/ethnic group might attenuate these disparities [61]. However, the underlying causes for the decreasing trend in endometrial cancer incidence among women 50–69 years old from 2016 to 2021 are unclear and warrant further investigation.

The COVID-19 pandemic profoundly disrupted healthcare systems, leading to a temporary decline in cancer screenings, diagnoses, and elective medical procedures [16]. With the rapid spread of SARS-CoV-2 in early 2020, healthcare organizations and government agencies prioritized pandemic response efforts, recommending the suspension of nonessential medical visits and elective surgeries to conserve healthcare resources and reduce virus transmission. As a result, routine gynecologic evaluations, diagnostic imaging, and biopsies were delayed, directly impacting endometrial cancer detection rates [62,63,64,65]. Fear of potential virus exposure in healthcare settings also discouraged many individuals from seeking routine medical care, exacerbating the decline in cancer screenings. The disruption of cancer screening services delayed cancer detection and diagnosis [16], as indicated by the subsequent decrease in detected cases for that time period. This delay not only led to a temporary drop in the incidence rates from 2019 to 2020 but also had long-term clinical consequences. It resulted in the subsequent detection of cancer at more advanced stages, corroborated by our results showing an increased prevalence of metastasis among diagnosed cancer cases in the first year of the COVID-19 pandemic. Unlike other malignancies, endometrial cancer typically presents with noticeable symptoms, such as abnormal vaginal bleeding [66]. Such a symptom often prompts individuals to quickly seek medical attention, increasing the chances of early detection. The observed temporary decrease in cancer incidence rates during the COVID-19 pandemic can be linked to the disruption of routine medical services and cancer screening. Our findings stress the importance of maintaining public access to essential healthcare services to ensure the timely diagnosis and treatment of cancer, even during a major public health crisis. Developing resilient healthcare infrastructures, implementing telemedicine-based triage systems, and reinforcing patient outreach efforts will be crucial for mitigating the impact of future global health emergencies on cancer diagnosis and treatment outcomes.

A major strength of our study is that we used data from the USCS database, which provides comprehensive, population-based cancer surveillance covering the entire US population. This large and diverse dataset provides a robust foundation for the study’s findings. The USCS database enhances the statistical power and generalizability, enables us to perform more precise estimates, and increases the reliability of observed trends such as the incidence rates among various age groups and racial/ethnic groups in this study. Additionally, our study benefits from the integration of BRFSS data collected via a large nationally-representative survey [21] to accurately adjust endometrial cancer trends according to hysterectomy prevalence.

One limitation of this study is the lack of information on key risk factors for endometrial cancer, including obesity, polycystic ovary syndrome, and hormone replacement therapy, within the USCS database. These factors play a crucial role in endometrial carcinogenesis, and their absence limits our ability to analyze the impact of these risk factors and assess their specific contributions to endometrial cancer trends over time. In addition, there are no data on molecular features for The Cancer Genome Atlas (TCGA) categorization [8] of endometrial cancers, which have greater prognostic values compared to traditional histologic classifications, in the dataset used in our analyses [67]. Our study also did not account for changes in screening practices, improvements in early detection [12], and shifts in healthcare utilization patterns that might influence endometrial cancer rates over the study period. Without accounting for these external influences, it becomes challenging to attribute changes solely to intrinsic factors. Given the limitations inherent to the study’s methodology and the characteristics of the data source, establishing definitive conclusions about the underlying causes proves challenging. Additionally, APCs for the trends are based on time periods indicated by the observed rates rather than on predetermined intervals. Consequently, these APCs should be interpreted with caution, particularly regarding their statistical significance.

## 5. Conclusions

In conclusion, our findings highlight evolving trends in endometrial cancer incidence among both premenopausal and postmenopausal women, emphasizing the dynamic nature of the disease’s epidemiology. Notably, we observed an increasing incidence among younger (20–49 years) and older (70+) women, while a declining trend was noted among women aged 50–69 years from 2016 to 2021, a pattern that warrants further investigation. Additionally, the COVID-19 pandemic led to a temporary decline in diagnosed cases from 2019 to 2020 across all age groups, coinciding with healthcare disruptions.

## Figures and Tables

**Table 1:** The case counts and adjusted incidence rates of endometrial cancer in 2019–2021 among adult women in the United States by age group, histologic type, and race/ethnicity.

	2019		2020		2021	
	Count	Rate (95% CI)	Count	Rate (95% CI)	Count	Rate (95% CI)
Age group						
20–49	6985	115.4 (112.7–118.1)	6147	103.4 (100.8–106.0)	6784	113.8 (111.1–116.5)
50–69	31,338	1045.0 (1033.4–1056.6)	28,089	947.6 (936.5–958.7)	30,289	1012.8 (1001.4–1024.2)
70+	15,947	1385.3 (1363.8–1406.8)	15,225	1284.8 (1264.5–1305.3)	16,558	1339.4 (1319.1–1359.9)
Histologic grade						
Low risk	34,380	337.1 (333.5–340.6)	30,525	302.5 (299.1–305.9)	32,927	323.3 (319.8–326.8)
High risk	8279	81.2 (79.4–82.9)	7410	73.4 (71.8–75.1)	7579	74.4 (72.8–76.1)
Histologic type						
Type 1	42,724	418.9 (414.9–422.9)	38,279	379.3 (375.5–383.1)	41,688	409.4 (405.4–413.3)
Type 2	6468	63.4 (61.9–65.0)	6071	60.2 (58.7–61.7)	6442	63.3 (61.7–64.8)
Rural/Metro						
Metro	45,808	518.8 (514.1–523.6)	41,603	475.1 (470.5–479.7)	44,861	507.6 (503.0–512.4)
Non-metro	7296	532.1 (519.9–544.4)	6518	488.4 (476.6–500.3)	6910	513.0 (501.0–525.2)
Race/Ethnicity						
NHW	33,974	539.0 (533.3–544.8)	30,400	492.4 (486.9–497.9)	31,964	516.4 (510.8–522.1)
NHB	7150	548.5 (535.8–561.3)	6776	523.5 (511.1–536.1)	7421	562.5 (549.7–575.3)
NHAIAN	365	459.3 (413.1–507.3)	375	463.1 (417.0–510.7)	366	446.4 (401.6–492.9)
NHAPI	2485	314.2 (301.9–326.6)	2299	285.6 (274.0–297.4)	2690	333.4 (320.9–346.1)
Hispanic	7719	441.0 (431.2–450.9)	6856	386.7 (377.6–395.9)	7875	433.9 (424.3–443.5)
Region of residence						
Northeast	9924	509.3 (499.3–519.3)	9110	465.8 (456.2–475.4)	10,004	512.4 (502.4–522.5)
Midwest	11,875	568.6 (558.4–578.9)	9864	519.5 (509.2–529.7)	10,510	551.1 (540.6–561.6)
South	19,408	522.3 (515.0–529.7)	18,098	481.5 (474.5–488.5)	19,449	507.9 (500.8–515.0)
West	11,780	480.3 (471.7–489.0)	10,928	440.0 (431.8–448.3)	11,667	467.0 (458.5–475.5)

Rate: cases per 1,000,000 persons. Rates were adjusted for hysterectomy prevalence and age adjusted to the 2000 US standard population. CI: confidence interval; NHW: non-Hispanic White; NHB: non-Hispanic Black; NHAIAN: non-Hispanic American Indian/Alaska Native; NHAPI: non-Hispanic Asian or Pacific Islander.

## Data Availability

The data used in this study are from the United States Cancer Statistics public use database, which is publicly available through the Centers for Disease Control and Prevention (CDC) website, which has established procedures for accessing the data. We will direct interested parties to the appropriate contacts to request access. Methods for our analysis will be made available upon request.
